# Immunogenicity of chondrocyte sheets: a review

**DOI:** 10.3389/fimmu.2025.1529384

**Published:** 2025-03-07

**Authors:** Juncen Li, Huilin Sun, Jiaqi Guan, Bohui Li, Chen Jin, Shanhong Xie, Yu Liu

**Affiliations:** ^1^ College of Clinical Medicine, Shandong Second Medical University, Weifang, China; ^2^ Department of Plastic and Reconstructive Surgery, Shanghai 9th People’s Hospital, Shanghai Jiao Tong University School of Medicine, Shanghai Key Laboratory of Tissue Engineering, Shanghai, China; ^3^ National Tissue Engineering Center of China, Shanghai Jiao Tong University, Shanghai, China; ^4^ Shanghai Resthetic Biotechnology. Co., Ltd, Shanghai, China

**Keywords:** cartilage, cell sheets, immunity, inflammation, tissue engineering

## Abstract

The chondrocyte sheet is a sheet-like cell structure obtained by separating *in vitro* expanded and fused autologous chondrocytes from the bottom of the culture dish by physical means. The cell sheet contains autologous chondrocytes, extracellular matrix secreted by chondrocytes, and connective structures established between cells and matrix, and between cells and cells. In cartilage tissue engineering, chondrocyte sheets technology has great potential for the treatment of cartilage defects. Chondrocyte sheets have a low immunogenicity because they avoid the immune reaction caused by scaffolding materials. However, chondrocyte sheets can still cause severe local tissue swelling in the short term after implantation, resulting in a poor patient experience. In individual cases, an inflammatory reaction may even occur, leading to resorption of the chondrocyte sheet. This may be immunogenetically related to chondrocyte membrane surface-associated antigens, components of the extracellular matrix secreted by chondrocytes, and various bioactive components in the culture medium used during *in vitro* chondrocyte culture. Therefore, in order to investigate the causes of local tissue swelling and immune-inflammatory reactions induced by the implantation of chondrocyte sheets, this article reviews the immunogenicity of chondrocyte-associated antigens, components of the extracellular matrix of cartilage, and the active components of the cell culture medium.

## Background

Lacking vascular and nerve nourishment, cartilage has a very limited regenerative capacity and is difficult to self-repair once the damage is caused (1). In recent years, with the rapid development of tissue-engineered chondrocyte sheet technology, chondrocyte sheets are now able to successfully construct tissue-engineered cartilage tissues and be used for the treatment of cartilage defects (2)

**Figure 1:**
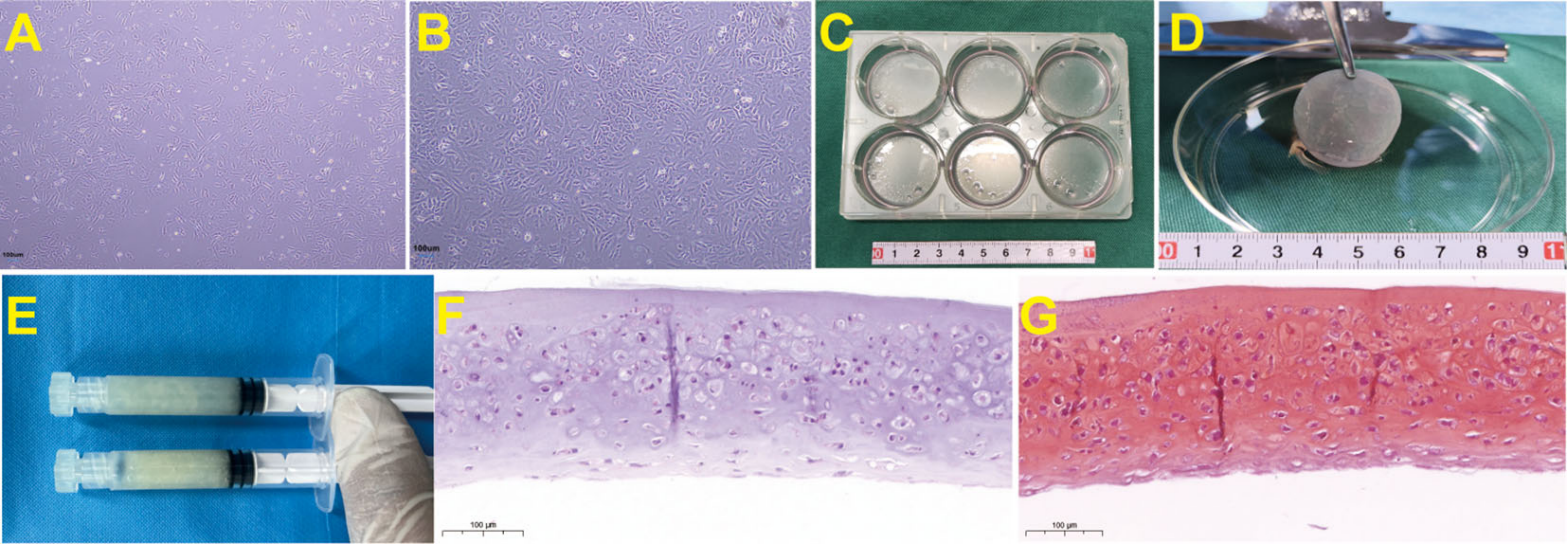
Preparation process and histological staining of goat ear chondrocyte sheets. **(A)** Primary culture of goat ear chondrocytes shows good adhesion to the wall under the microscope, with cells forming spindle or polygonal shapes. **(B)** Goat ear chondrocytes fill the bottom of the culture dish, with enlarged chondrocytes that secrete extracellular matrix. **(C)** After chondrocytes are amplified and passaged to P3 passages, they are high-density inoculated into a 6-well plate and continue to be cultured until the cell membrane is formed. **(D)** After culturing the chondrocyte in a 6-well plate for 1 month, the cell sheet was mechanically separated from the bottom of the 6-well plate using a cell scraper. The obtained chondrocyte sheet has a semi transparent sheet like appearance. **(E)** The chondrocyte sheet is physically shredded to prepare a granular chondrocyte sheet, which is then placed in a syringe for later use. **(F)** HE staining of chondrocyte sheets before implantation(40×). **(G)** Safranin O-Fast Green Staining of chondrocyte sheets before implantation (40×).

**Figure 2:**
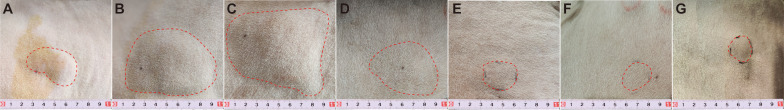
Granulated goat ear chondrocyte sheets (dose: 1ml) was injected into autologous subcutaneous tissue, causing local swelling reaction in a short period of time. On the second day after injection, the swelling range reached its maximum, and then gradually decreased. the red dashed line represents the swelling range. **(A)** Immediate implantation of chondrocyte sheets subcutaneously. **(B)** On the 1st day after implantation of chondrocyte sheets subcutaneously. **(C)** On the second day after implantation of chondrocyte sheets subcutaneously. **(D)** On the 3rd day after implantation of chondrocyte sheets subcutaneously. **(E)** On the 4th day after implantation of chondrocyte sheets subcutaneously. **(F)** On the 5th day after implantation of chondrocyte sheets subcutaneously. **(G)** On the 6th day after implantation of chondrocyte sheets subcutaneously.

The chondrocyte sheet is a sheet-like cell structure obtained by physically separating *in vitro* expanded and fused autologous chondrocytes from the bottom of the culture dish. This cell sheet contains autologous chondrocytes, extracellular matrix secreted by chondrocytes, and connective structures established between cells and matrix, and between cells and cells (**Figure 1**). Chondrocyte sheets are capable of forming very homogeneous, large-volume cartilage tissue in the subcutaneous environment of large, immunologically sound animals such as goats (3). Chondrocyte sheets are clinically safe and effective in the treatment of cartilage defects in the knee joint (4). However, the current problem with chondrocyte sheets is that they can trigger severe local tissue swelling in the short term after implantation, resulting in a poor patient experience (**Figure 2**). In individual cases, there is a possibility of an inflammatory reaction leading to resorption of the chondrocyte sheet. This may be immunogenetically related to chondrocyte membrane surface-associated antigens, components of the extracellular matrix secreted by the chondrocytes, and a variety of biologically active components in the medium used during *in vitro* chondrocyte culture (5).

Therefore, in order to investigate the causes of localized tissue swelling and immunoinflammatory reactions triggered by the implantation of chondrocyte membrane slices, the immunogenicity of chondrocyte-associated antigens, components of the extracellular matrix of cartilage, and active components of the cell culture medium are reviewed in this article.

## Cell sheets technology

In 1990, Yamada et al. (6) covalently bound poly-N-isopropyl acrylamide (PIPAAm) to the bottom of a cell culture dish for the first time, and controlled the adhesion and detachment of the cells from the bottom of the dish by modulating the temperature change, thus obtaining a technique that allows cell detachment and passaging culture without the need for trypsin digestion of the cells. The principle of this technology mainly relies on the temperature-sensitive material property of PIPAAm, i.e., cells can adhere to the Petri dish when the culture temperature is >32°C. As the temperature decreases, PIPAAm gradually changes from hydrophobic to hydrophilic, and the cells are separated from the bottom of the Petri dish. In 1993, Okano et al. (7) were the first to report the success of this technique in obtaining intact and viable cell sheets. The cell sheets contained cells and extracellular matrix without scaffolding material, thus avoiding the series of immunogenicity and safety of scaffold degradation products caused by exogenous scaffolds (7), and achieved good therapeutic results in dilated cardiomyopathy and ischemic cardiomyopathy-induced severe heart failure (8, 9). Since then, subsequent scholars have continued to try to prepare various tissue cell sheets using various types of cells to construct tissue-engineered bone, cartilage, blood vessels, myocardium, cornea, pancreatic islets, and other tissues (10–13), which have been used to treat lesions such as bone defects, cartilage defects, corneal defects, and post-esophagectomy defects (2, 14). Currently, the cell sheet technology has been widely used in the field of cartilage tissue engineering, and a very good series of results have been achieved. Shimizu et al. (15) reported that *in vivo* transplantation of chondrocyte sheets could promote TGF-β expression and repair of cartilage damage. Ebihara et al. (16) applied chondrocyte sheets to repair total cartilage defects of the knee joints in pigs, and the results showed that the cell sheet group had significant repair effects. The repair effect was remarkable. However, in our experiments, we observed that chondrocyte sheets still triggered an inflammatory response after implantation without the involvement of scaffolding materials. We analyzed that this might be related to the immunogenicity of chondrocytes, extracellular matrix, and culture medium components.

## Immunogenic components of chondrocyte sheets

Chondrocyte sheets contain chondrocytes and cartilage extracellular matrix. Studies have shown that many immunogenic substances exist in chondrocytes (17), such as human chondrocyte glycoprotein-39, chondrocyte autoantigens CH65, CD38, CD30, CD28, integrin-associated proteins, and major histocompatibility complexes MHC I, MHC II, etc. Especially with the *in vitro* cell cultures, as well as with the use of some culture medium components, which may change the natural chondrocyte cells’ original composition, leading to upregulation of immunogenic substances. There are many macromolecular proteins in the extracellular matrix secreted by chondrocytes, e.g., type II collagen, proteoglycan polymers, cartilage oligomeric matrix proteins, etc., and these proteins tend to be immunogenic, binding to B-cell and T-cell antigen receptors, stimulating cellular activation, proliferation, and differentiation, and the production of antibodies and sensitizing lymphocytes (18).

## Immunogenic components of chondrocytes

### Human cartilage glycoprotein-39

Human cartilage glycoprotein-39 (HC-gp39) is produced by chondrocytes, synovial fibroblasts, macrophages, and neutrophils, and is involved in tissue reconstruction and extracellular matrix degradation. The gene is located on chromosome 1, q32.1, and the protein structure is formed by the combination of heparin and chitinase. It is a highly conserved glycoprotein, also known as YKL-40 because of its amino-terminal initiation of three amino acids [tyrosine (Y), lysine (K), and leucine (L)], and a relative molecular weight of 40,000, and is homologous to mammalian chitinase ligase family members, but has no chitosanase activity itself (19, 20). Studies have shown that HC-gp39 peptide can be recognized by Peripheral Blood Mononuclear Cells (PBMC) in RA patients, causing PBMC proliferation and inducing chronic aggressive arthritis in BALB/c mice (21), and HC-gp39 can be highly expressed in articular cartilage and synovial membrane of RA patients, possibly participate in the immunopathological process of RA by being released locally in the joints, acting as an autoantigen to induce T cell activation, and promoting tissue reconstruction or matrix degradation. In the serum of RA patients, HC-gp39 was positively correlated with interleukin-6 (IL-6) and C-reactive protein (CRP), and it can regulate vascular endothelial growth factor, which plays an important role in inflammation, angiogenesis, cell proliferation and differentiation, extracellular matrix remodeling, and anti-apoptosis (22). This suggests that HC-gp39 may act as an autoantigen on chondrocyte sheets to induce an immune-inflammatory response in the body.

Studies have shown that HC-gp39 levels are significantly elevated when the body experiences diseases characterized by inflammation or tissue remodeling (23). In clinical practice, HC-gp39 can be expressed at a high level in the serum of active RA patients, and the increase of the expression level of HC-gp39 often indicates a serious degree of arthritis (24). In addition, the level of HC-gp39 is also significantly increased in patients with Takayasu arteritis, Kawasaki disease, pregnancy diabetes, early diabetes nephropathy, dermatomyositis, bronchial asthma, cervical cancer, endometrial cancer, systemic lupus erythematosus, primary Sjogren’s syndrome and other diseases (25–35). This suggests that HC-gp39 has important clinical significance in the diagnosis, occurrence, development, prognosis, and observation of therapeutic effects of diseases in clinical practice.

### Integrin-related proteins

Integrin-associated protein, also known as CD47, has a relative molecular mass of about 50 × 10^3^, is a highly glycosylated cell surface transmembrane protein, and a member of the immunoglobulin superfamily. It consists of an IgV-like structure with an amino-terminal extracellular variable region, a periplasmic region consisting of 3-5 highly hydrophobic periplasmic fragments, and a short selectively spliced hydrophilic carboxy-terminal cytoplasmic tail region (36). Signal-regulated protein alpha (SIRPα) is also known as SH2 domain⁃containing protein tyrosine phospha⁃tase substrate⁃1(SHPS⁃1), a transmembrane protein belonging to IgSF, is a typical heterogeneous immunoreceptor in the SIRP family. CD47 interacts with integrin proteins (e.g., αvβ3, αIIbβ3, and α2β1), and thrombospondin-1 (TSP⁃1) and acts as a SIRPα ligand. When CD47 binds to SIRPα on the macrophage surface, it phosphorylates two typical ITIM tyrosine residues at the tail end of SIRPα, which enables macrophages to exert “pro-phagocytic” or “anti-phagocytic” effects (37, 38). Studies have shown that chondrocytes express CD47, and *in vitro* culture and autologous subcutaneous transplantation as well as oxidative damage can lead to conformational changes of CD47 in chondrocytes, and the conformationally altered CD47 binds to SIRPα on the macrophage surface under the facilitation of TSP-1 binding, which triggers phagocytosis by macrophages. This may be one of the mechanisms by which a localized immune-inflammatory response, mainly involving macrophages, occurs after chondrocyte sheets are implanted in large animals (39).

Clinical studies have shown that CD47, as a natural immune checkpoint molecule, is highly expressed in cancer tissues such as gastric cancer, leukemia stem cells, Hodgkin’s lymphoma, breast cancer, cervical cancer, pancreatic cancer, and non-small cell lung cancer, and even the level of CD47 expression in tumor stem cells is higher than that in tumor cells (40–46). Research has shown that CD47 can evade tumor immunity, affect tumor progression and metastasis, and participate in processes such as cell apoptosis, proliferation, adhesion, and migration (47). Therefore, CD47 has also become an important target for studying human tumors. By using anti-CD47 antibodies to block the CD47-SIRP α pathway, it mediates cell phagocytosis and targets tumor cells, providing a new therapeutic strategy for treating human malignant tumors (48). However, researchers also face significant challenges posed by this target, such as the challenge of blood toxicity in drug design and clinical dose tolerance, as well as the challenge of selecting animal models for evaluating drug efficacy and safety (49). Therefore, further research and development of drugs targeting CD47 antibodies is still needed.

### Major histocompatibility complexes

Major Histocompatibility Complex (MHC) is a multigene family located on chromosomes, characterized by tight interlocking and polymorphism, and its expression products exist on the surface of a variety of cells, which constitutes the material basis of transplantation rejection, and plays an important role in the regulation of cellular recognition and immune response, which is closely related to the human immune response and disease resistance (50). There are three main classes of MHC, namely MHC I, MHC II, and MHC III, and their expression products are cell surface transmembrane proteins, called MHC antigens, that bind protein fragments of host and pathogen origin. MHC molecules have a different distribution of expression between species. In mouse chondrocytes, MHC I, MHC II, CD4 molecules, and S-100 protein are expressed (50); in rabbit chondrocytes, MHC I and MHC II class I molecules are expressed (51); and in human chondrocytes, only MHC I molecules are expressed, and CD4 and MHC II molecules are not detected (52). The main function of MHC molecules is to bind and deliver antigenic peptides to CD8+ and CD4+ T cells for their recognition, and the antigen receptors of CD8+ and CD4+ T cells are specific for antigenic peptides delivered by MHC molecules.MHC class I molecules are responsible for endogenous antigenic peptide recognition and delivery, and proteasomically processed peptides in the endoplasmic reticulum bind to MHC class I molecules to form peptide-MHC class I molecules, and then the complex is delivered to the cell surface, activating CD8+ T cells, and activated CD8+T cells are converted into active cytotoxic T lymphocytes (CTLs), which kill target cells. MHC II molecules are mainly involved in the delivery of exogenous antigenic peptides to CD4+ T cells, causing CD4+ helper T cells (Th) to activate, proliferate, and express corresponding lymphokines, triggering the body to react with the peptide. lymphokines, which triggers the body to initiate a humoral immune response (53). Almost all cell membranes express MHC class I molecules, and it has been shown that MHC class I molecules in chondrocytes are located in the cytoplasm around the nucleus of the cell, which may be related to the lower immunogenicity of chondrocytes (54). MHC class II molecules are generally expressed on the surface of immunopresenting cells, and their expression on the surface of other cells, such as tumor cells, is known as induced expression. The induction of MHC class II molecules has been well-studied in Oncology, inducible expression of MHC class II molecules increases the immunogenicity of tumor cells and causes recognition and antigen presentation of tumor cells by macrophages. Studies have shown that porcine chondrocytes induced by IFN-γ can express MHC class II antigens during *in vitro* culture, which can significantly stimulate lymphocyte proliferation (52). This suggests that chondrocytes may be induced to express their own MHC antigens with changes in the extracellular environment during *in vitro* culture, thereby increasing the immunogenicity of chondrocytes. This may be a relevant factor for autologous chondrocyte membrane sheet reimplantation to trigger the body to develop immune inflammation.

### Chondrocyte membrane proteins

Studies have shown the presence of specific anti-chondrocyte membrane antibodies (ACMA) in the sera of RA and OA patients, which are highly specific and bind only to chondrocyte surface proteins (CH65, CD38, CD30, CD28) and not to collagen cell membranes, fibroblast membranes, and pancreatic tumor cell membranes. cell membranes and pancreatic tumor cell membranes. This antibody has significantly higher ACMA in osteoarthritis and rheumatoid arthritis sera than in normal controls (17). This suggests that the chondrocyte autoantigens CH65, CD38, CD30, and CD28 may be involved in the immune response process as autoantigens of chondrocyte sheets.

It has been reported that CD38 is a membrane-bound multifunctional protein that was first identified as a surface marker in lymphocytes, functioning both as a cell surface-expressed receptor and as an enzyme (55). CD38 can be widely expressed on the surface as well as in the tissues of many immune populations, including CD4+ cells, CD8+ cells, B lymphocytes, and natural killer cells, among others. CD38 as a receptor can bind to CD31 on the surface of T cells, leading to T cell activation and the production of a variety of cytokines. CD38 also has enzymatic activity that catalyzes the generation of cyclic adenosine diphosphate ribose (cADPr) from NAD +, which in turn facilitates Ca2 + release. When the metabolism of NAD and its CD38 enzyme are dysfunctional, rheumatic diseases including systemic sclerosis, systemic lupus erythematosus, and rheumatoid arthritis are induced (56). Therefore, inhibition of CD38 enzyme activity may be a promising therapeutic target. In addition, studies have shown that CD38 also plays an important role in bone remodeling, mainly involved in the regulation of osteoclast formation and bone resorption. The expression of CD38 in human knee cartilage increases with age, which is closely associated with the development of age-related spontaneous OA. The results of Jinjin Ma et al. (55) showed that CD38 inhibitor treatment not only prevents articular cartilage degeneration but also prevents subchondral osteosclerosis, which opens up the possibility of utilizing CD38 to prevent the development of OA. CD38 expression has also been associated with inflammatory mediator-activated pathways, such as NF-κB and MAPK. Paulo Gil Alabarse et al. (56) demonstrated that CD38 could be upregulated in human knee OA chondrocytes and chondrocytes stimulated by the pro-inflammatory cytokine IL-1β, and its overexpression resulted in reduced cellular NAD/NADH levels and enhanced catabolic response to IL-1β. Following joint injury in CD38-deficient mice, synovial inflammation as well as pain in the joints were reduced, as were subchondral bone changes. The above suggests that CD38 can participate in the body’s immune response as an autoantigen on the chondrocyte membrane. In addition, CH65, a 65-kDa chondrocyte protein that is a component of chondrocyte autoantigens, can also be used as a target antigen in the Lewis rat adjuvant arthritis model to participate in the pathogenesis of arthritis, and it can be used in immune reactions with T cells and antibodies in rats. Pretreatment of rats with CH65 or Mycobacterium hsp65, but not human hsp60, significantly delayed the development of adjuvant arthritis in Lewis rats (17). This suggests that CH65 may be a potential autoantigen in the pathogenesis of adjuvant arthritis in Lewis rats and that it may also be a relevant factor in the induction of immune inflammation in the body by chondrocyte sheets.

## Cartilage extracellular matrix immunogenicity components

### Type II collagen

Type II collagen (Col-II) is mainly synthesized and secreted by chondrocytes and constitutes a major component of the extracellular matrix of cartilage, which plays a crucial role in the development and maturation of chondrocytes (57). Col-II accounts for more than 80% of the dry weight of decellularized cartilage extracellular matrix (ACECM), and the structure of the collagen network formed determines the mechanical properties of cartilage (58). Col-II is a macromolecular protein with a highly conserved amino acid sequence and a relatively low degree of variation among different species (59). Col-II molecule consists of three identical α1 chains, each containing 1487 amino acids, with a molecular weight of about 130 KDa (depending on the species origin) (60). The three α1 chains form a left-handed helix by themselves, and then further intertwine to form a right-handed three-stranded superhelix structure, which is a major and unique structural region of the collagen molecule and is called the “triple helix domain”. The most characteristic feature of the triple helix is the [Gly-X-Y]_n_ periodic repeating arrangement of amino acids, where the positions of X and Y are usually proline (Pro) and hydroxyproline (Hyp), respectively (61). Each chain has a short peptide extension at the end of the helix, i.e. telopeptides (amino (N)- and carboxyl (C)-telopeptides), which are non-helical and do not contain the [Gly-X-Y]n repeat sequence (62). The telopeptide structural domains determine intermolecular interactions that contribute to and stabilize normal fiber assembly. The amino acid sequence of telopeptides varies from species to species, while certain cross-linking regions involved in fiber formation are highly conserved. Once secreted into the extracellular matrix, collagen molecules are linked head to tail in a four-molecule-one staggered arrangement, which in turn covalently cross-links into collagen fibers through polymerization and disulfide bonds, forming the skeleton of the cartilage matrix.

It has been reported that Col-II is immunogenic and capable of inducing arthritis when emulsified with adjuvants (63). Anti-Col-II autoantibodies were also found in the sera of patients with rheumatoid arthritis, suggesting that Col-II induces autoimmune responses of an arthritic nature *in vivo* in T and B cells (64). It has been found that the antigenic epitopes determining the immunogenicity of Col-II are divided into three main categories: (1) those located at the non-helical end of the collagen molecule (telopeptides), which are present in both natural and denatured collagens; (2) those amino acid sequences in the helical region of the α1-chain, which need to be unserialized to be exposed and are thus present in denatured collagens; (3) those induced by the triple-helical spatial conformation, which must preserve the helical structural integrity, and thus are present only in natural collagens. The triple-helical domain of Col-II is highly conserved among species, and the amino acid sequence does not vary by more than a few percent. Of particular interest are the antigenic epitopes located in the helical region of the collagen molecule hidden in the amino acid sequence, which binds to antibodies to induce an immune response when the antigenic epitopes are exposed after de-helicalization (65). In addition, Col-II has been found to have an immunomodulatory effect by inhibiting the STAT1 signaling pathway in pro-inflammatory macrophages to alleviate degeneration of osteoarthritic articular cartilage matrix (66); it also induces macrophage polarization to M2, and activated M2 macrophages express pro-chondrocytic cytokines to stimulate the secretion of matrix components from chondrocytes, and activate the glycine receptor and reduce intracellular calcium concentration to inhibit chondrocyte apoptosis and hypertrophy (67). This suggests that Col-II may be another important factor in triggering the development of immune inflammation in the body after autologous reimplantation of chondrocyte sheets.

In clinical practice, the degradation and metabolism of type II collagen is accelerated when articular cartilage is degenerated or damaged (68). In the presence of various proteases such as matrix metalloproteinases (MMPs), type II collagen can be broken down into peptide fragments such as carboxyl-terminal 3/4 fragment of type II collagen (Col-3/4), 1/4 helical peptide (HELI-II), carboxyl-terminal telopeptide of type II collagen (CTX-II), and other peptide fragments, which become biomarkers of cartilage metabolism during OA (69). It has been shown that the level of Col-3/4 in urine is positively correlated with the Kellgren-Lawrence imaging grading of the knee joints in KOA patients, and the detection of the level of Col-3/4 in urine can be used to assess the condition of KOA patients (70). In addition, other studies have shown that CTX-II concentration has the highest correlation with osteoarthritis symptoms and the degree of joint damage closely, especially the degree of knee joint space narrowing and the degree of subchondral osteosclerosis have the highest correlation with CTX-II concentration, and the monitoring of CTX-II concentration in urine has the diagnostic value of OA (71). The above suggests that type II collagen and its breakdown products are closely related to cartilage inflammation, and their detection in the clinic may provide better diagnostic, therapeutic, and prognostic measures for patients with degenerative cartilage diseases. It also provides a new direction for clinical assessment of the degree of body inflammation triggered by autologous chondrocyte sheets.

### Proteoglycan polymer

Proteoglycan polymer (aggrecan) is the main macromolecular proteoglycan component of the cartilage matrix, there are multiple functional domains of the template proteoglycan, and the core protein has three spherical regions, consisting of G1, G2, and G3, each domain contains cysteine residues and is connected by hydrogen bonding, G1 and G2 are separated by interglobular domains, there is a glycosaminoglycan (GAG) attachment region between G2 and G3, which is rich in chondroitin sulfate (CS) and keratan sulfate (KS). G1 is located at the amino-terminal end of the core protein and can be divided into three functional domains, A, B1, and B2, the B-type domains interact with hyaluronic acid (HA); G2 also has two B-type domains, but they do not interact with HA, and its function is still unclear; G3 is located at the carboxyl terminus of core proteins and contains a variety of specialized structural domains that are indispensable for normal post-translational processing of proteoglycan core proteins and subsequent proteoglycan secretion. The GAG attachment region consists of three domains that can attach CS and KS. Proteoglycan molecules are not isolated in the extracellular matrix but exist as aggrecan (72). Each aggrecan consists of a central hyaluronic acid chain and 100 proteoglycan molecules emanating from it, which are stabilized by linker protein connections. The large aggrecan molecules are wrapped in collagen scaffolds in the tissue thus stabilizing the proteoglycan in the extracellular matrix (72). Rarely is aggrecan present in cartilage in its intact form; instead, it is replaced by core proteins subjected to extracellular proteolytic processing, which ultimately produces aggrecan that is not an intact proteoglycan molecule (73). There are many biomolecules involved in aggrecan metabolism, and the two most important enzymes in degradation metabolism are matrix metalloproteinases (MMPs) and polyproteoglycans (74), whereas the most important ones in anabolism are tissue inhibitor of metalloproteinases (TIMP) (75) and α2 macroglobulin (76). The interactions and constraints among these four biomolecules maintain the metabolic homeostasis of proteoglycans.

Studies have shown that aggrecan can activate peripheral blood T cells from RA patients to induce autoimmune responses, and the main T cell epitope that induces arthritis is present in the G1 globular domain of the antigen, where the main peptide epitope, amino acids 280-292, induces an IgG2-type antibody response in addition to T cell proliferation and interferon(IFN-γ)secretion. The presence of peptides in the G1 region produced by degradation of aggrecan in the synovial fluid of patients with early RA suggests that aggrecan may be involved in the induction and maintenance of autoimmune responses in RA (77).

In addition, clinical studies have shown a significant correlation between serum aggrecan levels and the occurrence of lumbar disc herniation (78), with patients with lumbar disc herniation having significantly lower serum aggrecan levels than the healthy population, and this study even found that serum aggrecan also differed significantly among patients with different clinical efficacy, with treatment-naïve lumbar disc herniation patients having serum aggrecan levels were even lower (79). It is well known that the inflammatory response is an important factor in the development of lumbar degenerative disease, and the production and accumulation of inflammatory factors promotes extracellular matrix degradation and exacerbates the loss of aggrecan expression (80). This suggests that the expression level of serum aggrecan in patients can indirectly assess the degree of inflammation associated with degenerative cartilage lesions in the organism. This is an important guideline for observing the inflammatory state of the organism triggered by autologous chondrocyte sheets in the clinic.

### Cartilage oligomeric matrix protein

Cartilage Oligomeric Matrix Protein (COMP) with the family of Thrombospondins (TSPs), also known as Thrombospondin-5 (TSP-5), is an important protein in the composition of the extracellular matrix (ECM). Mörgelin (81) (1992) isolated and characterized it for the first time from Swarm rat chondrosarcoma, and it is mainly found in cartilage, bone, skin, synovial membrane, tendon, and ligament tissues (82), and can be secreted and produced by a variety of cells such as chondrocytes, tendonocytes, myofibroblasts, cardiomyocytes, vascular smooth muscle cells, platelets, etc. (83). COMP is a multi-structural glycoprotein, with five subunits with a molecular mass of about 100 ku linked together by disulfide bonds to form a pentameric monomeric form with a molecular mass of about 524 ku, containing an amino-terminal complex helical domain (NTD), four epidermal growth factor-like repeats (EGF-like), eight type III calmodulin-like repeats (Type III), and a globular C-terminal domain (CTD) (84). The hollow composite helical structure of NTD can bind and accommodate a variety of hydrophobic molecules, such as vitamin D3, retinoic acid, etc. (85). The COMP produced by cell secretion can carry signaling molecules such as vitamin D3 to cartilage tissues after entering the humoral circulation, which in turn promotes chondrocyte proliferation, hypertrophy, and new bone formation in an endochondral osteogenic manner (86), meanwhile, NTD, as a pentameric polymeric structural domain, connects with adjacent subunits through disulfide bonds, so that the five monomers present a bouquet-like arrangement. the EGF-like domain can be linked to a variety of metalloproteinases, such as matrix metalloproteinase-13 (MMP-13), a disintegrin-like and metalloproteinase with thrombospondin motifs (ADAMTS-4, ADAMTS-7, ADAMTS-12), etc., which mediate the degradation of COMP proteins and participate in the regulation of the dynamic equilibrium between the synthesis and catabolism of the ECM in tissues (84). The Type III structural domain contains 13 calcium ion binding sites, and the appropriate calcium ion concentration is essential for the folding and correct conformation of COMP proteins (84). With the special bouquet arrangement of COMP, CTD can bind to a large number of ECM components such as I, II, IX, X, XII, collagen type, fibronectin, proteoglycan, matrillin-1, matrillin-3, matrillin-4, etc. (87, 88), which provides more space for the assembly of multiple ECMs, and the huge network organism formed by them then provides a good physical support for tissues and improves their tolerance to mechanical stress, while CTDs can transmit signals and participate in cell growth regulation by binding to cell surface adhesion molecules, such as integrin α5β1, α7β1, αVβ3, CD47 and other extracellular matrix receptors (88).

In addition, this region can also bind to certain growth factors and cytokines to promote the function of the latter, such as binding to transforming growth factor (TGF)-β and bone morphogenetic protein (BMP-2, BMP-4), etc. to participate in the corresponding downstream signaling and regulation of cell proliferation and differentiation (89). As an ECM secreted by chondrocytes, COMP interacts with type II collagen and other ECM components such as proteoglycans to stabilize articular cartilage structure. A large number of clinical studies have shown that the expression levels of a variety of proteases that degrade ECM components are elevated in articular cartilage in early OA, such as MMP-13, which degrades Col- II, and proteoglycans ADAMTS-4, ADAMTS-5, etc. (90), whereas the major degrading proteases of COMP, such as ADAMTS-4, ADAMTS-7, ADAMTS-12, MMP-1, MMP-9, MMP-19, MMP-20, etc. are up-regulated, leading to increased COMP degradation, which can further destabilize cartilage ECM structure, leading to loss of other ECM and accelerating the progression of OA (91, 92). Studies have shown that higher levels of COMP-complement C3b complexes can be detected in serum as well as synovial fluid of RA patients, suggesting that COMP can activate the human complement system via the bypass pathway and bind C1q and mannan-binding lectin (MBL), thus inhibiting the classical and lectin pathways of complement activation. The release of degradation fragments of COMP into the joint cavity may activate complement in the synovial fluid and further exacerbate the destruction of articular cartilage; whereas the failure to detect COMP-C3b complexes in the sera of patients with OA suggests that the degradation fragments produced by COMP may be different in the 2 types of arthritis, and that binding to complement may require some specific amino acid sequences or antigenic epitopes to be realized (93). Smith et al. (94) used mass spectrometry to analyze COMP degradation fragments in OA cartilage, and found that COMP degradation exposed some neoantigenic epitopes that some full-length proteins do not possess, and in the future, if we can comprehensively analyze the neoantigenic epitopes of COMP degradation fragments in arthritis, it will be helpful in the application and development of COMP as a molecular marker for arthritis.

## Immunogenic components of culture medium for *in vitro* cell culture

Cell culture medium is an important factor in *in vitro* cell culture, which can affect cell growth. The cell culture medium can be divided into natural medium, synthetic medium, and serum-free(SF) medium (95). Currently, synthetic media are more commonly used cell culture media, such as DMEM, MEM, RPMI1640, etc. Before being used for cell culture, almost all synthetic media need to be added with natural liquids, such as fetal calf serum(FCS), because the growth of the majority of animal cells depends on the presence of serum, and the majority of the cells cannot proliferate without serum in the ordinary culture media. However, FCS is not a simple liquid, it is an extremely complex mixture of many kinds of plasma proteins, fats, carbohydrates, inorganic substances and other biomolecules of different sizes, as far as proteins are concerned, there are no fewer than 150 kinds of protein components contained in the serum, and some FCS even contain immunoglobulin IgG1 and IgM, therefore, FCS residues in biologics can easily cause allergic reactions to the serum of vaccinated individuals (96). This was confirmed by the study of Keitaro Ohmori et al. (97), who identified the presence of IgE against FCS in the sera of dogs that developed allergic reactions after vaccination. Immunoblotting analyses yielded that several biologically active components of FCS, including albumin, could act as allergens and cause allergic reactions in dogs after vaccination. Therefore, we believe that the shift from FCS-containing medium to fully chemically defined serum-free (SF) medium is a trend for the clinical application of autologous chondrocyte sheets, but the use of SF medium for culturing the cells, although eliminating the problems of xenogeneic contamination and batch-to-batch variations, brings many new limitations, for example, the chondrocytes are difficult to attach to the wall, the proliferation slow proliferation rate, etc. Kang et al. (98) also showed that MSCs cultured with SF differed significantly from those cultured with FCS in terms of morphology, surface markers, senescence status, differentiation ability and senescence/apoptosis status. In the rat osteochondral defect model, SF-cultured MSCs showed poorer cartilage repair. This suggests that it is crucial that the chondrogenic capacity of SF-cultured MSCs should be pre-determined when selecting SF medium for the fabrication of chondrocyte sheets for clinical cartilage repair.

## Immune status of clinical individual differences

In the clinic, patients with cartilage defects often have mechanical wear and tear or autoimmune diseases, which can further expose the chondrocyte autoantigens HC-gp39, CD38, and CH65 and stimulate the body’s immune response. Some studies have shown that when the organism develops rheumatoid arthritis, degenerative changes in articular cartilage, rheumatoid arthritis, and other diseases characterized by inflammation or tissue reconstruction, the expression levels of chondrocyte-associated antigens HC-gp39, CD38, and so on, are markedly elevated (23, 24). The presence of inflammation promotes the degradation of cartilage extracellular matrix, aggravates the loss of aggrecan expression (80), degrades type II collagen into various peptide fragments such as carboxy-terminal 3/4 fragment of type II collagen (Col-3/4), 1/4 helical peptide (HELI-II), carboxy-terminal crosslinked telopeptide of type II collagen (CTX-II) (68). Inflammation also exposes and degrades COMP, which in turn activates the body’s complement system, further exacerbating the destruction of articular cartilage (93). If a patient undergoes autologous chondrocyte sheets implantation in this disease state, it will likely result in the development of immune inflammation in the body, increasing the risk of chondrocyte sheets implantation failure. Therefore, the assessment of the patient’s physical condition is another important factor that should be considered in advance for clinical implantation of autologous chondrocyte sheets.

## Conclusions

Chondrocyte sheets have great potential to treat cartilage defects. However, chondrocyte sheets can cause severe local tissue swelling in the short term after implantation, resulting in a poor patient experience. In some cases, an inflammatory reaction may even occur, leading to the resorption of the chondrocyte membrane, which greatly hampers the promotion of the clinical application of chondrocyte sheets. Therefore, it is important to explore the immunogenicity of chondrocyte sheets. Detection of the antigens associated with chondrocyte sheets in the clinic will help predict the degree of inflammation caused by chondrocyte sheets, as well as the assessment of the clinical effect of chondrocyte sheets after autologous reimplantation. It can even guide clinicians to provide individualized interventions for patients, reduce the risk of complications associated with chondrocyte sheets implantation, and improve the quality of clinical care. Currently, the immunogenicity of human chondrocyte glycoprotein-39, chondrocyte autoantigens CH65, CD38, CD30, CD28, integrin-associated proteins, major histocompatibility complexes MHC I and MHC II, as well as type II collagen, proteoglycan polymers, and COMP in the chondrocyte extracellular matrix and their reactive signaling pathways have been extensively studied, but the chondrocyte membrane sheets of other potential antigens still need to be further explored. In addition, we need to consider the problems of xenogeneic contamination and batch-to-batch variation caused by fetal bovine serum medium. In conclusion, we expect more innovative methods to be applied to chondrocyte sheets, and realize the wide clinical application of chondrocyte sheets at an early date.
